# Covid-19: Perspectives on Innate Immune Evasion

**DOI:** 10.3389/fimmu.2020.580641

**Published:** 2020-09-30

**Authors:** Nima Taefehshokr, Sina Taefehshokr, Nima Hemmat, Bryan Heit

**Affiliations:** ^1^Department of Microbiology and Immunology, Center for Human Immunology, The University of Western Ontario, London, ON, Canada; ^2^Immunology Research Center, Tabriz University of Medical Sciences, Tabriz, Iran; ^3^Robarts Research Institute, London, ON, Canada

**Keywords:** SARS-CoV-2, macropahge, innnate immune, MHC trafficking, cytokine - immunological terms

## Abstract

The ongoing outbreak of Coronavirus disease 2019 infection achieved pandemic status on March 11, 2020. As of September 8, 2020 it has caused over 890,000 mortalities world-wide. Coronaviral infections are enabled by potent immunoevasory mechanisms that target multiple aspects of innate immunity, with severe acute respiratory syndrome coronavirus 2 (SARS-CoV-2) able to induce a cytokine storm, impair interferon responses, and suppress antigen presentation on both MHC class I and class II. Understanding the immune responses to SARS-CoV-2 and its immunoevasion approaches will improve our understanding of pathogenesis, virus clearance, and contribute toward vaccine and immunotherepeutic design and evaluation. This review discusses the known host innate immune response and immune evasion mechanisms driving SARS-CoV-2 infection and pathophysiology.

## Introduction

In December 2019, the outbreak of novel severe acute respiratory syndrome coronavirus 2 (SARS-CoV-2) – the causative agent of Coronavirus disease 2019 (COVID-19) – began in Wuhan, Hubei Province, China, from where it spread rapidly. On March 11, 2020 the World Health Organization declared COVID-19 a global pandemic (1, 2). COVID-19 is not the first severe respiratory disease outbreak caused by coronaviruses (CoVs) in humans, with two epidemic diseases – severe acute respiratory syndrome (SARS-CoV) in 2003–2004 and Middle East respiratory syndrome (MERS–CoV) in 2012 – caused by similar zoonotic transmission of CoVs (3). The primary symptoms of COVID-19 are similar to those of SARS-CoV and MERS-CoV: fever, fatigue, dry cough, discomfort in the upper chest, occasional diarrhea, and dyspnea. Severe cases exhibit secondary infections, cytokine-storm driven sepsis, and multi-organ failure (4, 5). COVID-19 patients primarily develop pneumonia, lymphopenia, and feature pulmonary ground glass opacity on chest CT (6, 7).

Coronaviruses are in the *Coronaviridae* family, enveloped viruses with a positive-sense single-stranded RNA genome ranging from 26 to 32 kb in size (8). In humans CoV infections are common, with four CoVs (229E, NL63, OC43, and HKU1) causing ∼10% of common cold cases (4). The infection of human cells by CoVs is mediated by interactions between envelope-anchored spike glycoprotein (S-protein) of CoV with one of two host cell receptors: angiotensin-converting enzyme 2 (ACE2) or CD147 (9, 10). The S-protein consists of two subunits: S1 which functions as the receptor-binding domain (RBD), and S2 which drives the fusion of the viral membrane with the host cell membrane (11). Spike glycoprotein activation and viral entry is mediated by cleavage of the S protein by the host transmembrane protease, serine 2 (TMPRSS2) (12, 13). Sequencing of SARS-CoV-2 from patients revealed that it shares 79.6% homology to SARS-CoV, 50% MERS-CoV, and 96% to bat SARS-like CoV at the whole genome level (12, 14). The RBD of SARS-CoV-2 is derived from a pangolin-infecting CoV and exhibits a 10-fold increase in affinity between the RBD and ACE2 compared to SARS-CoV, further consistent with ACE2 as the prominent receptor for SARS-CoV-2 (15). This increase in receptor affinity was generated by the recombination of the pangolin CoV and a bat SARS-like CoV within the RBD region, a characteristic which could lead to a more efficient cell entry (16). Interestingly, crytal structure evaluation by cryo-electron microscopy (Cryo-EM) showed that SARS-CoV-2 RBD is biased toward the lying state conformation, which reduces receptor binding by burying the RBD within the spike protein trimer. In contrast, the SARS-CoV RBD is mostly in the exposed “standing up” state which favors receptor binding (17–19). This bias toward the lying state may favor SARS-CoV-2 immune evasion by masking the RBD domain from neutralizing antibodies. There have been reports that SARS-CoV-2 can also gain entry into cells via CD147, but the importance of this pathway for viral entry, and the concordant receptor-binding motifs, remain largely unelucidated (10).

Less well understood than SARS-CoV-2’s biology is it resulting immune responses, immunopathology, and immune evasion mechanisms. Understanding these responses will be vital for the development of immunotherapies or vaccines against COVID-19 (1, 20). Coronaviruses are adept at manipulating immune responses and interfere with the interferon (IFN) pathway, with several structural proteins (M and N) and non-structural protein (NSP1 and NSP3) from SARS-CoV and MERS-CoV acting as interferon antagonists (21). These CoVs also interfere with pattern recognition receptor (PRR) signaling such as Toll-like receptors (TLRs) and retinoic acid-inducible gene I (RIG-I) like receptors (22), and generate a strong inflammatory response (23, 24). This drives a non-productive inflammation, resulting in a cytokine storm and disseminated damage to the host, while avoiding induction of an anti-viral interferon response. Indeed, an early study of 41 COVID-19 patients identified increased levels of pro-inflammatory cytokines including IL-2 and IL-7, with more severe disease producing elevated G-CSF, MCP-1, MIP-1α, IP-10, and TNF-α (25). These pro-inflammatory cytokines drive an influx of neutrophils and other myeloid cells into the lung, producing a strong local inflammatory response and significant immunopathology (26). This is consistent with SARS and MERS, indicating that a cytokine storm and lymphopenia play a crucial role in the COVID-19 pathogenesis (25, 27, 28). In addition to manipulating cytokines, CoVs also manipulate other immunological processes including antigen presentation (29). In this review we discuss the major innate immunological pathways involved in responses to CoV infection, and the mechanisms used by SARS-CoV-2 and related CoVs to overcome these defenses.

## Interferons and Cytokines

The innate immune system is an evolutionary conserved set of cellular and chemical defenses critical for the recognition and restriction of pathogens, and for the subsequent activation of an adaptive immune response (21). Innate immunity is initiated by pathogen-associated molecular patterns, evolutionarily conserved molecular structures specific to pathogens that act as ligands for PRRs. Ligand binding triggers signaling pathways that coverage on transcription factors including NF-κB, IRF3, and AP-1, which synergistically promote type I interferon (IFN-I) production. These cytokines act in a paracrine fashion on neighboring cells via the IFN-α/β receptor (IFNAR) and induce expression of interferon-stimulated genes (ISGs) (30). Interferon-stimulated genes are an essential component of innate antiviral defense, acting to both limit viral entry and restricting viral replication after a virus enters a host cell (31, 32). Interferon-stimulated gene expression is driven predominantly by IFNAR-mediated activation of the Jak/STAT pathway, resulting in binding of STAT1 homodimers and STAT1/2 heterodimers to the promoter region of ISGs.

Severe acute respiratory syndrome coronavirus 2 has evolved multiple mechanisms to manipulate this key antiviral response. It was recently demonstrated that ACE2 is an ISG, suggesting that SARS-CoV-2 may exploit IFN-driven ACE2 upregulation to enhance infection (31). Furthermore, CoVs including SARS-CoV and MERS-CoV encode multiple proteins which antagonize IFN signaling. These functions represent a key anti-immune mechanism of SARS-like CoV’s, and are critical for the viral manipulation of the innate immune response and promoting early viral pathogenesis (33, 34). Because SARS-CoV-2 may utilize ISG’s to enhance infectivity, and because it is unclear whether the IFN response restricts SARS-CoV-2 replication, it is unknown if IFN-directed therapies will be beneficial to COVID-19 patients. A recent study found that COVID-19 patients who failed to produce IFN-α experienced more severe clinical outcomes (35). In addition, IFN-α potently inhibits the replication of SARS-CoV-2 *in vitro* (36). The National Health Commission of China has proposed guidelines for the treatment of SARS-CoV-2, which includes aerosolized recombinant IFN-α, based on the observation that IFN-α inhibits SARS-CoV replication *in vitro* (37, 38). At the time of this writing only one retrospective analysis of these treatment guidelines has been published, identifying a modest benefit of IFN-α in combination with lopinavir, ritonavir, and ribavirin (39). The combinatorial use of these drugs makes it difficult to assess the role of IFN-α alone, but these data suggest that induction of the IFN-I pathway produces a beneficial response in SARS-CoV-2 patients. In addition to IFN-I, type III IFNs (IFN-λ) exhibit more potent antiviral functions than IFN-α in treating influenza infection, without activating inflammation and tissue damage induced by IFN-α (40, 41). On the other hand, it has been shown that IFN-λ treatment could inhibit bacterial uptake by neutrophils in the lung during influenza superinfection, suggesting that IFN-λ may increase susceptibility to lower repiratory tract infection, potentially increasing the risk of super-infection of COVID-19 patients to super-infection with other pathogens (42, 43).

Similar to type I IFNs, IFNs-λ is decreased during COVID-19 infection (44), and IFN-λ hinders SARS-CoV-2 replication *in vitro* in human intestinal epithelial cells (45). Interestingly, IFN-λ is present in lower airways in COVID-19 patients where it mediates antiproliferative effects in during the repair of the lung epithelium. This antiprolifertative effect occurs via p53 induction, but due to its slowing of epithelial repair, may increase the risk of life-threating bacterial superinfections in the lung during both influenza and COVID-19 infection (46, 47). Thus, timing and duration are critical parameters of IFN action and in COVID-19 patients who contract a secondary bacterial infection, it might be crucial to intervene with recombinant interferons when virus is in upper airways. Later in disease, when inflammation is increased in the lower airways, it may be beneficial to block signaling cascades initiated by interferons and other inflammatory cytokines in order to control superinfecting pathogens.

The mechanisms used by CoVs to manipulate the IFN response can be divided into three categories: (1) avoidance, where the virus protects itself from recognition by PRRs, (2) IFN induction suppression, where the virus inhibits the transcription of interferons (48), and (3) IFN signaling suppression, where viral proteins inhibit IFNAR signaling (49). The viral membrane (M) protein, nucleocapsid (N) protein, and the non-structural proteins NSP1, NSP3b, NSP4a, NSP4b, NSP15, play crucial roles in modulation of the host immune response (50). The SARS-CoV N protein modulates signaling though the TGF-β receptor by forming a complex with Smad3. This enhances Smad3/p300 transcription, driving lung fibrosis. This interaction also prevents Smad3 from complexing with Smad4, thereby enhancing the survival of infected cells by antagonizing TGF-β-sensitized apoptosis (51). Moreover, SARS-CoV and MERS-CoV hide their RNA genome from host detection by cytosolic (e.g., RIG-I) and endosomal (e.g., TLR3/7) PRRs by replicating in double membrane vesicles that excludes these PRRs (52, 53). Indeed, both the induction of IFN-α or β and the ability to restrict both MERS-CoV and SARS-CoV infection requires TLR3 signaling, and SARS-CoV is able to antagonize the TLR signaling pathway via its papain-like protease (PLpro) (54, 55). Finally, during viral replication the SARS-CoV and MERS-CoV N protein catalytically modifies host proteins through SUMOylation and ubiquitination, via interactions with the host proteins hUbc9 and TRIM25, respectively (22, 56). While the targets of N protein-mediated SUMOylation remains unclear, N protein-targeted ubiquitination targets RIG-I for proteasomal degradation, thus depleting the cell of a critical virus-detecting PRR (22). The N proteins of SARS-CoV and SARS-CoV-2 are 90% conserved, suggesting that these immune avoidance activities are likely conserved in SARS-CoV-2 (Supplementary Figure 1). Thus, through manipulating multiple components of the host’s PRR system, CoVs are able to limit or avoid activation of many host anti-viral processes.

**FIGURE 1:**
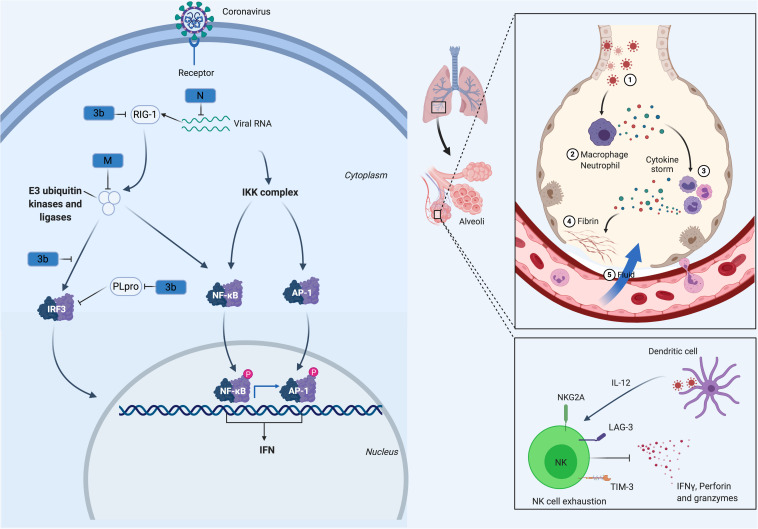
CoV Innate Immune Evasion. The innate immune response to CoV’s is activated upon detection of viral pathogen-associated molecular patterns, such as double-stranded RNA, via host PRRs such as RIG-I. Following viral recognition, transcription factors including NF-κB, AP-1 and IRF3 are activated and translocate to the nucleus where they induce the expression of interferons. Both MERS-CoV and SARS-CoV, through their M, N, non-structural proteins (NSP1, 3b, 4a, 4b, 5, 6), and PLpro, have developed mechanisms to interfere with these signaling pathways. This alters the cytokine secretion profile of infected cells to enhance the recruitment of myeloid immune cells over NK cells, which in turn produce more cytokines, creating a cycle of inflammation that damages the lung. Many of these processes are likely conserved in SARS-CoV-2.

Analysis of MERS-CoV patients with differing severity has demonstrated significantly lower IFN-I responses in patients who succumb to infection versus to those who recover, highlighting the importance of IFN induction (57). It has been shown that the IFN pathway is inhibited by SARS-CoV-2 to an extent similar to the inhibition by MERS-CoV and SARS-CoV, thereby impairing both innate T cell antiviral responses (58–60). While the specific molecular pathways mediating the suppression of IFN induction in SARS-CoV-2 have not been fully elucidated, these mechanisms are well understood in SARS-CoV and MERS and are likely conserved (Figure 1). Severe acute respiratory syndrome coronavirus NSP1 promotes the degradation of IFN-β mRNA (61), while ORF6 disrupts IFN induction by preventing the transport of IRF3 and STAT1 into the nucleus (62; 63). This occurs via two mechanisms, with ORF6 and ORF3b reducing IFNAR signaling by disrupting STAT1 nuclear import and promoting STAT1 proteolytic degradation, respectively (62, 64). ORF4a interacts with dsRNA and the RIG-I like receptor cofactor PACT, inhibiting IFN induction (65, 66). ORF4b blocks IFN induction by binding to both TBK1 and IKKε (67, 68). MERS-CoV’s ORF4b, 5, and M proteins have been shown to prevent nuclear translocation of IRF3 (69). At least some of these pathways are conserved in SARS-CoV-2, with a recent study demonstrating that SARS-CoV-2 ORF3b is a potent IFN antagonist, which suppresses IFN induction more efficiently than the SARS-CoV ortholog (70). Notably, a recent study reported that ORF3b is one of the most common antibody-recognized antigens during early stage COVID-19 infection (71), suggesting that ORF3b is highly expressed in the acute stage of the infection and may represent an immunodominant epitope. NSP13, NSP14, NSP15, and ORF6 have been suggested to function as IFN antagonists by suppressing IRF3 nuclear localization (72). Additionally, SARS-CoV-2 M protein through interacting with RIG-I/MDA-5-MAVS signaling pathway inhibits the production of type I and III IFNs (73). As discussed above, the SARS and MERS PLpro is a deubiquitinating/deISGylating enzyme (74), which enhances immunoevasion by downregulating IFN-β transcription (75). The PLpro proteins of SARS-CoV and SARS-CoV-2 are 76% conserved, suggesting that this activity is likely conserved in SARS-CoV-2 (Supplementary Figure 2).

COVID-19 patients experience a cytokine storm that drives much of the pathophysiology of the disease. Both pro-and anti-inflammatory circulating cytokines increase in SARS-CoV-2 patients, with cytokines including G-CSF, TNFα, MCP1, IL-10, IL-2 and IL-7 becoming elevated in critically ill patients (76, 77). IL-6 is thought to be a major driver of pathology, with the highest levels observed in non-survivors and critically ill COVID-19 patients (78, 79). The mechanism leading to elevated IL-6 in severe COVID-19 is not currently clear, but is likely driven through activation of virus-specific PRRs. However, other pathways are likely involved in patients with severe disease. For example, SARS-CoV triggers the production of oxidized phospholipids, which then drives IL-6 production via TLR4 (80). The upregulation of IL-6 is an established effect of TLR4 signaling, which occurs via NF-κB and MAPK signaling pathways (81). Finally, the N protein of SARS-CoV-2 induces the expression of IL-6 in infected airway epithelium by binding to the NF-κB regulatory elements of the IL-6 promoter (82). Clearly, SARS-CoV-2 pathology is driven by an unbalanced cytokine response, although the viral processes driving these responses remain to be fully elucidated.

## Pattern Recognition Receptor Evasion

In addition to modulating IFN-I and IL-6 responses, CoVs also engage in immune evasion through limiting PRR activation. Coronaviruses replicate in a double-membrane structure derived from the endoplasmic reticulum (52). The endoplasmic reticulum lacks PRR’s, and as such, this compartment secludes the replicating virus from both cytosolic PRRs such as RIG-I and from endosomal PRRs such as TLR3 and TLR7 (83–85). In addition to physically excluding PRRs from the site of viral replication, CoVs suppress PRR signaling directly. As discussed above, coronaviral N-proteins direct the host cell ubiquitination machinery to target the cytosolic RNA sensor RIG-I, leading to RIG-I degradation (22). Coronaviruses also suppress TLR signaling; for example, the MERS-CoV spike protein induces expression of the negative TLR regulator IRAK-M in macrophages through activating the dipeptidyl-peptidase 4 receptor (86). Likewise, SARS-CoV induces the broad dysregulation of TLR-associated signaling molecules in infected macrophages, although the specific mechanism of this dysregulation and its net effect on TLR signaling has not been established (87). While these activities have not yet been reported in SARS-CoV-2, the high degree of conservation between it, MERS-CoV, and SARS-CoV indicates that some manipulation of the PRR system likely occurs during SARS-CoV-2 infection.

## Neutrophils

Neutrophilia is an early indicator of SARS-CoV-2 infection, although it is unclear whether this increase is due to the release of the marginated neutrophil pool versus the release of bone marrow-derived cells (88–90). While the extent to which neutrophils are responsible for COVID-19 pathophysiology is unclear, significant neutrophil infiltration has been reported in autopsied COVID-19 patients (91, 92). Infiltration is not the only mechanism by which neutrophils may cause pathology on COVID-19 patients. Indeed, pathological effects of neutrophil extracellular traps (NETs) have been identified in a variety of inflammatory conditions including thrombosis, sepsis, and respiratory failure (93–95). Neutrophil extracellular traps are comprised of extracellular DNA fibers, histones, microbicidal proteins, proteases such as neutrophil elastase, and oxidant enzymes such as myeloperoxidase, that are released by neutrophils in response to many infectious agents. If not regulated properly, NETs initiate and propagate inflammation and thrombosis (96–98).

For the first time, a recent study showed that NETs released by neutrophils contributes to organ damage and mortality in COVID-19 patients (99). This is consistent with another recent study that identified markers of NET release, including myeloperoxidase-DNA and citrullinated histone H3, in COVID-19 patients, with the sera of these patients potently inducing NETosis of control neutrophils *in vitro* (100). The high levels of IL-6 observed in COVID-19 patients is likely a driver of this NETosis, as in other inflammatory diseases IL-6 induces the systemic release of NETs (101, 102). Additional triggers of NETosis include virus-damaged epithelial cells (103, 104), activated endothelial cells (105), activated platelets (106, 107), and inflammatory cytokines such as IL-1β (108, 109). Transcriptional analysis of bronchoalveolar lavage fluid and peripheral blood mononuclear cells from COVID-19 patients showed that elevated levels of CXCL2 and CXCL8 contributes to the recruitment of neutrophils to the lung, aggravating the inflammatory response (110). Moreover, activated neutrophils express properdin, factor B, and C3, thus driving complement activation (111), a marker of severe COVID-19 disease (112, 113). Consistent with a role for NETs in COVID-19 immunopathology, a small clinical trial demonstrated a protective effect of dipyridamole, an FDA approved drug which inhibits NETosis via blocking signaling through adenosine A_2__*A*_ receptors (93, 114).

## NK Cells

Natural killer (NK) cells are essential to the control of viral infections, and functional impairment of NK cells correlate with persistence of SARS-CoV-2 (115, 116). Reduced peripheral blood NK cells numbers is frequently observed in severe COVID-19 patients (89, 116–118). Killer-immunoglobulin like receptors (KIRs) which are expressed on plasma membrane of NK cells alongside CD16 play crucial roles in NK cells licensing and their subsequent cytotoxic functions (119). In peripheral blood, NK cells expressing KIRs and CD16 are significantly decreased in SARS-CoV and SARS-CoV-2 infection, suggesting either impaired maturation of NK cells or migration of circulating NK cells into the peripheral tissues of SARS-CoV-2 patients (120, 121). Moreover, lower NK cell numbers correlate with higher IL-6 plasma concentrations in SARS-CoV-2 infection (117, 120). *In vitro*, IL-6 and soluble IL-6 receptor impair perforin and granzyme B production by healthy donor NK cells, which could be restored by treatment with the IL-6R inhibitor tocilizumab (122). Furthermore, secretion of cytokines such as IL-12 by macrophages and dendritic cells (DCs) could promote NK cell proliferation, cytotoxicity, survival, and IFN-γ production (123, 124). The later may occur through IFN-λ-mediated IL-12 production by macrophages (125), suggesting that early IFN-γ production and NK cell stimulation may act to limit SARS-CoV-2 infection.

The expression of inhibitory receptor NKG2A on NK cells results in the functional exhaustion of NK cells in chronic viral infection and cancer (126, 127). It is reported that NK cells become functionally exhausted in SARS-CoV-2 patients, as evidenced by increased NKG2A expression and decreased expression of CD107a, IFN-γ, IL-2 and granzyme B in NK cells (76, 116). Additionally, recent studies reported upregulated expression of the genes encoding inhibitory receptors including TIM3 and LAG3 in NK cells from COVID-19 patients (60, 128). The implications of this high incidence of exhausted NK cells in SARS-CoV-2 patients remain unelucidated. While NK cell numbers and activity appear to be diminished in COVID-19 patients, anti-S protein antibodies are capable of inducing NK-cell mediated antibody-dependent cell cytotoxicity in an *in vitro* model of SARS-CoV-2 infection, indicating that normally functional NK cells should contribute to protective immunity against SARS-CoV-2 (129).

## Macrophages

Macrophages are one of the primary drivers of innate immunity in response to CoV infection, with macrophage activity driving both inflammation, and much of the pathology, in COVID-19 patients (130). Macrophages in the lung and upper respiratory tract act as sentinel cells and are among the first immune cells to encounter incoming virions. In response, these macrophages can limit early viral replication through initiating a IFN-I response, as well as through initiating an inflammatory response to recruit additional immune cells (131). While this inflammatory response is required to initiate immune responses against SARS-CoV-2, excess inflammation in the form of a cytokine storm contributes to the mortality associated with COVID-19 (132). The lung has at least two distinct macrophage populations - interstitial macrophages and alveolar macrophages (AMs) (125), and there are at least two subsets of interstitial macrophages – of which, the nerve-and airway-associated macrophages (NAMs) may be particularly important for restricting inflammation in response to CoV’s. Nerve-and airway-associated macrophages are highly divergent from AMs in their gene expression profile and tissue localization. Nerve-and airway-associated macrophages are located in the lung interstitia, closely associated with innervating nervous tissue, and have a different ontology and growth factor dependence than AMs (133). During influenza infection NAMs are critical in limiting viral-induced inflammation, whereas AMs are pro-inflammatory and required for viral clearance. These findings indicate that AMs are anti-viral and pro-inflammatory, whereas NAMs are and act to prevent excessive and damaging inflammation. Importantly, during influenza NAMs suppress IL-6 production, indicating that NAMs may be a critical control point in determining IL-6 levels, and thus may regulate the cytokine storm in COVID-19 patients (133).

Consistent with these observations, distinct macrophage populations were identified in the lungs of COVID-19 patients, and while this study did not investigate NAMs, it did identify enrichment of anti-inflammatory monocyte-derived (FCN1^*high*^) macrophages in patients with mild disease, while resident pro-fibrotic (SPP1^*high*^) and inflammatory AM (FAPB4^+^) populations dominated in patients with severe COVID-19 (134). Combined, these studies demonstrate that macrophage polarization and the relative proportion of macrophage subtypes is a major factor in COVID-19 severity.

Macrophages can be infected by SARS-CoV-2 (130), indicating that SARS-CoV-2 directly manipulates macrophages to evade immunity. It is unclear what effect SARS-CoV-2 infection has on macrophage function, but infection of macrophages by other CoVs is known to induce altered functional states. Macrophages infected by MERS-CoV express high levels of major histocompatibility complex I (MHC I), CD80 and CD86, but lack major histocompatibility complex II (MHC II), indicating that MHC II presentation is impaired by MERS-CoV (58, 135). Consistently, MHC II downregulation was recently demonstrated in monocytes and B cells from COVID-19 patients (128). Moreover, monocyte HLA-DR is downregulated in severe COVID-19 patients, with expression patially restored by an IL-6 inhibitor (136). The mechanism of MHC II downregulation is not completely understood, but is due in-part to changes in the epigenetic landscape of the infected cells (137). Indeed, the epigenetic downregulation of MHC II is a mechanism shared by other CoVs, for example, the human CoV-EMC also downregulates MHC II via epigenetic reprograming (29). However, epigenetic reprograming of antigen presentation is not universal, and for example, is not a feature of SARS-CoV despite this virus also limiting antigen presentation on MHC II (29).

The recently published interactome of SARS-CoV-2 provides some potential insights into mechanisms by which this virus may interfere with macrophage function (138). The blockade of interferon signaling, described above, would limit ISG expression in macrophages, including cytokine-induced MHC II expression (139). The SARS-CoV-2 protein Nsp5 interacts with the epigenetic regulator histone deacetylase 2 (HDAC2), which regulates MHC II expression and cytokine production (138, 140, 141). While it is unknown whether SARS-CoV-2 inhibits or enhances HDAC2 activity, this interaction indicates a potential direct modulation of the cytokine storm and antigen presentation. Nsp13 and ORF8 of SARS-CoV-2 interacts with multiple components of the Golgi trafficking system and may utilize this as a mechanism to restrict MHC export to the cell surface. Indeed, a recent study demonstrated that ORF8 of SARS-CoV-2 can directly bind to MHC I molecules at endoplasmic reticulum and redirect them to autolysosomes for degradation (142). This is a common approach used by viruses to limit antigen presentation; for example, the HIV Nef protein restricts antigen presentation on MHC I by redirecting MHC trafficking toward the Golgi (143, 144). Lastly, SARS-CoV-2’s Nsp10 interacts with the endocytosis regulator AP2, a critical regulator of MHC II trafficking to antigen loading compartments (138, 145, 146). While these immunoevasory mechanisms remain largely theoretical, the ability of SARS-CoV-2 to infect macrophages and interact with proteins central to macrophage function suggests a potent ability to modulate macrophage activity, and through this, the systemic immune response.

## Dendritic Cells

Dendritic cells are key players in antigen presentation, cytokine production, priming specific T cell responses, and a loss of DCs function could lead to delayed immune responses in COVID-19 patients (147). Previous studies demonstrated that SARS-CoV infects DCs, resulting in poor antiviral cytokine expression with an upregulation of inflammatory chemokines including MIP-1α (148). SARS-CoV also enhanced pro-inflammatory cytokine (IL-6 and IL-12) production by DCs in response to secondary activation signals by bacterial LPS, further contributing to a damaging inflammatory response (149). Moreover, plasmacytoid dendritic cells (pDCs) have been identified as a subset of DCs able to secrete large amounts of IFN I after contact with CoVs (150), although this is not universal – MERS-CoV infection induces significantly higher type I and III IFNs production by pDCs than does SARS-CoV (151). While the role of pDCs in COVID-19 remains largely unexplored, the high levels of IFN-Is produced by these cells suggests a protective role.

Severe acute respiratory syndrome coronavirus 2 infection appears to target DCs directly – a recent study identified reduces DC frequency and functionally in COVID-19 patients, and a concordant impairment in the subsequent activation of T cells. Thus, in addition to further driving the cytokine storm, SARS-CoV-2 may infect DCs to limit DC maturation, and thus suppress T cell-mediated responses (44, 152, 153). This may represent a critical juncture in disease progression, as interference of T cell activation by modulating DC maturation could account for the lack of long-lasting humoral immunity and other defects in adaptive immunity associated with CoV infection. While infection of DCs appears to play an important role in driving the cytokine storm and modulating T cell responsiveness to SARS-CoV-2, the specific mechanisms used by the virus to alter DC function remains to be investigated.

## Trained Immunity: A Defense Against COVID-19?

Recent studies have shown that innate immune populations may possess a memory phenotype, termed Trained Immunity (TRIM), wherein innate immune cells undergo mitochondrial, metabolic, and epigenetic reprograming following exposure to a pathogen (154), making the cell more responsive to subsequent pathogen exposures. Unlike with adaptive immunity, this “training” primes the cell to respond in an enhanced manner not only to the initial pathogen, but also to other pathogens encountered during the multi-week period over which TRIM lasts (154). TRIM can be effective against viral pathogens – for example, aerosolized bacterial lysates enhanced innate immune responses and increase survival against influenza A and other respiratory viruses (155, 156).

The induction of TRIM by Bacillus Calmette-Guerin (BCG) and other vaccines has been extensively demonstrated (157, 158). A recent study demonstrated that BCG administration to low-weight newborns reduced mortalities from infectious diseases by 43% over the neonatal period (159). It is tempting to postulate that the decreased incidence and rate of complications of COVID-19 reported in children (160) may be partially attributed to the frequent vaccinations – and therefore the frequent induction of TRIM – that occurs during routine childhood vaccination schedules. Encouraging studies have shown a correlation between universal BCG and influenza vaccination policies and reduced mortality rates in COVID-19 infection (161, 162), and while these studies are not directly indicative of a causal relationship between these vaccinations and protection against SARS-CoV-2, they suggest that using existing approved vaccines to induce TRIM may be a viable approach to limit the negative effects of COVID-19 (157, 158). Consequentially, a number of studies are investigating whether BCG and MMR vaccines can attenuate COVID-19 pathology (163–166).

While existing vaccines represent the quickest avenue to leverage TRIM as a preventative measure for COVID-19, other compounds may provide superior protection. For example, β-glucan polysaccharides found in the cell wall of bacteria, yeast and fungi, are known potent initiators of TRIM (167). β-glucans exert antiviral effects and decrease the severity of several respiratory viruses (168, 169). Orally administered β-glucan traffics into lymph nodes and spleen, where they activate DCs, leading to the expansion and activation of antigen-specific T cells and enhance T cell production of effector cytokines such as IFN-γ (170). This enhanced T cell activity results from β-glucan induced IFN-β production by DCs, which enhances the production of IFN-γ and Granzyme-B by CD8^+^ T cells (171). While β-glucans are commonly consumed by humans in foods prepared with yeast, care needs to be taken when considering their use for inducing TRIM in SARS-CoV-2 patients, as some studies have shown that β-glucans enhance M1 polarization of AMs – a phenomenon that may enhance the cytokine storm induced by SARS-CoV-2 (172, 173).

## Future Prospects and Conclusion

The rapid spread of SARS-CoV-2 has become a global concern. Currently, there are no approved drugs or vaccines to treat human CoVs, but recent advances in our understanding of the immune response and immune evasion mechanisms of CoV’s opens up many therapeutic avenues. These include mechanisms for limiting viral entry and replication, promoting viral clearance, and inducing productive anti-CoV immune responses. Investigating how SARS-CoV-2 modifies gene expression in innate immune cells will be crucial to identifying immune mechanisms that could be modulated to improve patient outcomes. Addressing IFN evasion mechanisms and preventing viral immune evasion may contribute to enhancing viral clearance and lessening immunopathology. While a major driver of the cytokine storm in COVID-19 patients, IL-6 has both pro- and anti-inflammatory properties, giving it a complex role in COVID-19 pathology. Inhibition of IL-6 signaling and elucidation of the mechanism which elevates IL-6 in patients will help to find new potential strategies to reduce pathology during COVID-19 infection. Finally, newly available tools such as next generation sequencing will provide key information on the clinical features of the disease and potential targets for the development of drugs and vaccines. While an effective treatment for COVID-19 remains elusive, this large array of tools and knowledge should enable the rapid development of preventative and therapeutic treatments for this newly emerged disease.

## Author Contributions

NT prepared the main body of the manuscript, with assistance from ST. NH performed the bioinformatics analysis. BH supervised the project and assisted in the authoring and revision of the manuscript. All authors contributed to the article and approved the submitted version.

## Conflict of Interest

The authors declare that the research was conducted in the absence of any commercial or financial relationships that could be construed as a potential conflict of interest.
