# Supplementary material for: Covid-19: Perspectives on Innate Immune Evasion
Source: Front Immunol. 2020 Sep 30;11:580641. doi: 10.3389/fimmu.2020.580641 (PMC7554241; doi:10.3389/fimmu.2020.580641)
Supplement: Supplementary file 1 [file Data_Sheet_1.PDF]

```
SARS-CoV-2    1 MSDNGPQ-NQRNAPRITFGGPSDSTGSNQNGERSGARSKQRRPQGLPNNTASWFTALTQH
SARS-CoV      1 MSDNGPQSNQRSAPRITFGGPTDSTDNNQNGGRNGARPKQRRPQGLPNNTASWFTALTQH

SARS-CoV-2   60 GKEDLKFPRGQGVPINTNSSPDDQIGYYRRATRRIRGGDGKMKDLSPRWYFYYLGTGPEA
SARS-CoV     61 GKEELRFPRGQGVPINTNSGPDDQIGYYRRATRRVRGGDGKMKELSPRWYFYYLGTGPEA

SARS-CoV-2  120 GLPYGANKDGIIWVATEGALNTPKDHIGTRNPANNAAIVLQLPQGTTLPKGFYAEGSRGG
SARS-CoV    121 SLPYGANKEGIVWVATEGALNTPKDHIGTRNPNNNAATVLQLPQGTTLPKGFYAEGSRGG

SARS-CoV-2  180 SQASSRSSSRSRNSSRNSTPGSSRGTSPARMAGNGGDAALALLLLDRLNQLESKMSGKGQ
SARS-CoV    181 SQASSRSSSRSRGNSRNSTPGSSRGNSPARMASGGGETALALLLLDRLNQLESKVSGKGQ

SARS-CoV-2  240 QQQGQTVTKKSAAEASKKPRQKRTATKAYNVTQAFGRRGPEQTQGNFGDQELIRQGTDYK
SARS-CoV    241 QQQGQTVTKKSAAEASKKPRQKRTATKQYNVTQAFGRRGPEQTQGNFGDQDLIRQGTDYK

SARS-CoV-2  300 HWPQIAQFAPSASAFFGMSRIGMEVTPSGTWLTYTGAIKLDDKDPNFKDQVILLNKHIDA
SARS-CoV    301 HWPQIAQFAPSASAFFGMSRIGMEVTPSGTWLTYHGAIKLDDKDPQFKDNVILLNKHIDA

SARS-CoV-2  360 YKTFPPTEPKKDKKKKADETQALPQRQKKQQTVTLLPAADLDDFSKQLQQSMS--SADST
SARS-CoV    361 YKTFPPTEPKKDKKKKTDEAQPLPQRQKKQPTVTLLPAADMDDFSRQLQNSMSGASADST

SARS-CoV-2  418 QA
SARS-CoV    421 QA

Total sites= 422 aa
Conserved sites= 382 aa
Variable sites= 37 aa
Deleted sites= 3 aa
```

Supplemental Figure 1: Protein Alignment of N-Protein from SARS-CoV and SARS-CoV-2. Alignment was performed using MEGA-X and aligned using ClustalW with the default settings. Conserved sites are highlighted in black; substitutions of biochemically similar amino acids are highlighted in grey. Alignment is with accession's SARS-CoV: NP_828858.1 and SARS-CoV-2: YP_009724397.2

```
SARS-CoV      1 APIKGVTFGEDTVWEVQGYKNVRITFELDERVDKVLNEKCSVYTVESGTEVTEFACVVAE
SARS-CoV-2    1 APTK-VTFGDDTVIEVQGYKSVNITFELDERIDKVLNEKCSAYTVELGTEVNEFACVVAD

SARS-CoV     61 AVVKTLQPVSDLLTNMGIDLDEWSVATFYLFDDAGEENFSSRMYCSFYPPDEEEEDDAEC
SARS-CoV-2   60 AVIKTLQPVSELLTPLGIDLDEWSMATYYLFDESGEFKLASHMYCSFYPPDEDEEE-GDC

SARS-CoV    121 EEEEIDETCEHEYGTEDDYQGLPLEFGASAETVRVEEEEEEDWLDDTTEQSE--------
SARS-CoV-2  119 EEEEFEPSTQYEYGTEDDYQGKPLEFGATSAALQPEEEQEEDWLDDDSQQTVGQQDGSED

SARS-CoV    173 ---------IEPEPEPTPEEP-------VNQFTGYLKLTDNVAIKCVDIVKEAQSANPMV
SARS-CoV-2  179 NQTTTIQTIVEVQPQLEMELTPVVQTIEVNSFSGYLKLTDNVYIKNADIVEEAKKVKPTV

SARS-CoV    217 IVNAANIHLKHGGGVAGALNKATNGAMQKESDDYIKLNGPLTVGGSCLLSGHNLAKKCLH
SARS-CoV-2  239 VVNAANVYLKHGGGVAGALNKATNNAMQVESDDYIATNGPLKVGGSCVLSGHNLAKHCLH

SARS-CoV    277 VVGPNLNAGEDIQLLKAAYENFNSQDILLAPLLSAGIFGAKPLQSLQVCVQTVRTQVYIA
SARS-CoV-2  299 VVGPNVNKGEDIQLLKSAYENFNQHEVLLAPLLSAGIFGADPIHSLRVCVDTVRTNVYLA

SARS-CoV    337 VNDKALYEQVVMDYLDNLKPRVEAPKQEEPPNTEDS--KTEEKSVVQKPVDVKPKIKACI
SARS-CoV-2  359 VFDKNLYDKLVSSFLEMKSEKQVEQKIAEIPKEEVKPFITESKPSVEQRKQDDKKIKACV

SARS-CoV    395 DEVTTTLEETKFLTNKLLLFADINGKLYHDSQNMLRGEDMSFLEKDAPYMVGDVITSGDI
SARS-CoV-2  419 EEVTTTLEETKFLTENLLLYIDINGNLHPDSATLVSDIDITFLKKDAPYIVGDVVQEGVL

SARS-CoV    455 TCVVIPSKKAGGTTEMLSRALKKVPVDEYITTYPGQGCAGYTLEEAKTALKKCKSAFYVL
SARS-CoV-2  479 TAVVIPTKKAGGTTEMLAKALRKVPTDNYITTYPGQGLNGYTVEEAKTVLKKCKSAFYIL

SARS-CoV    515 PSEAPNAKEEILGTVSWNLREMLAHAEETRKLMPICMDVRAIMATIQRKYKGIKIQEGIV
SARS-CoV-2  539 PSIISNEKQEILGTVSWNLREMLAHAEETRKLMPVCVETKAIVSTIQRKYKGIKIQEGVV

SARS-CoV    575 DYGVRFFFYTSKEPVASIITKLNSLNEPLVTMPIGYVTHGFNLEEAARCMRSLKAPAVVS
SARS-CoV-2  599 DYGARFYFYTSKTTVASLINTLNDLNETLVTMPLGYVTHGLNLEEAARYMRSLKVPATVS

SARS-CoV    635 VSSPDAVTTYNGYLTSSSKTSEEHFVETVSLAGSYRDWSYSGQRTELGVEFLKRGDKIVY
SARS-CoV-2  659 VSSPDAVTAYNGYLTSSSKTPEEHFIETISLAGSYKDWSYSGQSTQLGIEFLKRGDKSVY

SARS-CoV    695 HTLESPVEFHLDGEVLSLDKLKSLLSLREVKTIKVFTTVDNTNLHTQLVDMSMTYGQQFG
SARS-CoV-2  719 YTSN-PTTFHLDGEVITFDNLKTLLSLREVRTIKVFTTVDNINLHTQVVDMSMTYGQQFG

SARS-CoV    755 PTYLDGADVTKIKPHVNHEGKTFFVLPSDDTLRSEAFEYYHTLDESFLGRYMSALNHTKK
SARS-CoV-2  778 PTYLDGADVTKIKPHNSHEGKTFYVLPNDDTLRVEAFEYYHTTDPSFLGRYMSALNHTKK

SARS-CoV    815 WKFPQVGGLTSIKWADNNCYLSSVLLALQQLEVKFNAPALQEAYYRARAGDAANFCALIL
SARS-CoV-2  838 WKYPQVNGLTSIKWADNNCYLATALLTLQQIELKFNPPALQDAYYRARAGEAANFCALIL

SARS-CoV    875 AYSNKTVGELGDVRETMTHLLQHANLESAKRVLNVVCKHCGQKTTTLTGVEAVMYMGTLS
SARS-CoV-2  898 AYCNKTVGELGDVRETMSYLFQHANLDSCKRVLNVVCKTCGQQQTTLKGVEAVMYMGTLS

SARS-CoV    935 YDNLKTGVSIPCVCGRDATQYLVQQESSFVMMSAPPAEYKLQQGTFLCANEYTGNYQCGH
SARS-CoV-2  958 YEQFKKGVQIPCTCGKQATKYLVQQESPFVMMSAPPAQYELKHGTFTCASEYTGNYQCGH

SARS-CoV    995 YTHITAKETLYRIDGAHLTKMSEYKGPVTDVFYKETSYTTTIKPVSYKLDGVTYTEIEPK
SARS-CoV-2 1018 YKHITSKETLYCIDGALLTKSSEYKGPITDVFYKENSYTTTIKPVTYKLDGVVCTEIDPK

SARS-CoV   1055 LDGYYKKDNAYYTEQPIDLVPTQPLPNASFDNFKLTCSNTKFADDLNQMTGFTKPASREL
SARS-CoV-2 1078 LDNYYKKDNSYFTEQPIDLVPNQPYPNASFDNFKFVCDNIKFADDLNQLTGYKKPASREL

SARS-CoV   1115 SVTFFPDLNGDVVAIDYRHYSASFKKGAKLLHKPIVWHINQATTKTTFKPNTWCLRCLWS
SARS-CoV-2 1138 KVTFFPDLNGDVVAIDYKHYTPSFKKGAKLLHKPIVWHVNNATNKATYKPNTWCIRCLWS
```

```
SARS-CoV   1175 TKPVDTSNSFEVLAVEDTQGMDNLACESQQPTSEEVVENPTIQKEVIECDVKTTEVVGNV
SARS-CoV-2 1198 TKPVETSNSFDVLKSEDAQGMDNLACEDLKPVSEEVVENPTIQKDVLECNVKTTEVVGDI

SARS-CoV   1235 ILKPSDEGVKVTQELGHEDLMAAYVENTSITIKKPNELSLALGLKTIATHGIAAINSVPW
SARS-CoV-2 1258 ILKPANNSLKITEEVGHTDLMAAYVDNSSLTIKKPNELSRVLGLKTLATHGLAAVNSVPW

SARS-CoV   1295 SKILAYVKPFLGQAAITTSNCAKRLAQRVFNNYMPYVFTLLFQLCTFTKSTNSRIRASLP
SARS-CoV-2 1318 DTIANYAKPFLNKVVSTTTNIVTRCLNRVCTNYMPYFFTLLLQLCTFTRSTNSRIKASMP

SARS-CoV   1355 TTIAKNSVKSVAKLCLDAGINYVKSPKFSKLFTIAMWLLLLSICLGSLICVTAAFGVLLS
SARS-CoV-2 1378 TTIAKNTVKSVGKFCLEASFNYLKSPNFSKLINIIIWFLLLSVCLGSLIYSTAALGVLMS

SARS-CoV   1415 NFGAPSYCNGVRELYLNSSNVTTMDFCEGSFPCSICLSGLDSLDSYPALETIQVTISSYK
SARS-CoV-2 1438 NLGMPSYCTGYREGYLNSTNVTIATYCTGSIPCSVCLSGLDSLDTYPSLETIQITISSFK

SARS-CoV   1475 LDLTILGLAAEWVLAYMLFTKFFYLLGLSAIMQVFFGYFASHFISNSWLMWFIISIVQMA
SARS-CoV-2 1498 WDLTAFGLVAEWFLAYILFTRFFYVLGLAAIMQLFFSYFAVHFISNSWLMWLIINLVQMA

SARS-CoV   1535 PVSAMVRMYIFFASFYYIWKSYVHIMDGCTSSTCMMCYKRNRATRVECTTIVNGMKRSFY
SARS-CoV-2 1558 PISAMVRMYIFFASFYYVWKSYVHVVDGCNSSTCMMCYKRNRATRVECTTIVNGVRRSFY

SARS-CoV   1595 VYANGGRGFCKTHNWNCLNCDTFCTGSTFISDEVARDLSLQFKRPINPTDQSSYIVDSVA
SARS-CoV-2 1618 VYANGGKGFCKLHNWNCVNCDTFCAGSTFISDEVARDLSLQFKRPINPTDQSSYIVDSVT

SARS-CoV   1655 VKNGALHLYFDKAGQKTYERHPLSHFVNLDNLRANNTKGSLPINVIVFDGKSKCDESASK
SARS-CoV-2 1678 VKNGSIHLYFDKAGQKTYERHSLSHFVNLDNLRANNTKGSLPINVIVFDGKSKCEESSAK

SARS-CoV   1715 SASVYYSQLMCQPILLLDQALVSDVGDSTEVSVKMFDAYVDTFSATFSVPMEKLKALVAT
SARS-CoV-2 1738 SASVYYSQLMCQPILLLDQALVSDVGDSAEVAVKMFDAYVNTFSSTFNVPMEKLKTLVAT

SARS-CoV   1775 AHSELAKGVALDGVLSTFVSAARQGVVDTDVDTKDVIECLKLSHHSDLEVTGDSCNNFML
SARS-CoV-2 1798 AEAELAKNVSLDNVLSTFISAARQGFVDSDVETKDVVECLKLSHQSDIEVTGDSCNNYML

SARS-CoV   1835 TYNKVENMTPRDLGACIDCNARHINAQVAKSHNVSLIWNVKDYMSLSEQLRKQIRSAAKK
SARS-CoV-2 1858 TYNKVENMTPRDLGACIDCSARHINAQVAKSHNIALIWNVKDFMSLSEQLRKQIRSAAKK

SARS-CoV   1895 NNIPFRLTCATTRQVVNVITTKISLKGG
SARS-CoV-2 1918 NNLPFKLTCATTRQVVNVVTTKIALKGG

Total sites= 1948 aa
Conserved sites= 1477 aa (75.8%)
Variable sites= 442 aa
Deleted sites= 29 aa
```

Supplemental Figure 2: Protein Alignment of PLPro Protease from SARS-CoV and SARS-CoV-2. Alignment was performed using MEGA-X and aligned using ClustalW with the default settings. Conserved sites are highlighted in black; substitutions of biochemically similar amino acids are highlighted in grey. Alignment is with accession's SARS-CoV: NP_828862.2 and SARS-CoV-2: YP_009725299.1.
